# Experimental and Statistical Investigation to Evaluate Impact Strength Variability and Reliability of Preplaced Aggregate Concrete Containing Crumped Rubber and Fibres

**DOI:** 10.3390/ma15155156

**Published:** 2022-07-25

**Authors:** Packirisamy Swaminathan, Kothandapani Karthikeyan, Siva Ramakrishnan Subbaram, Jayaraman Sethuraman Sudharsan, Sallal R. Abid, Gunasekaran Murali, Nikolai Ivanovich Vatin

**Affiliations:** 1 Department of Civil Engineering, Mangalam College of Engineering, Ettumanoor 686631, India; sam7586@gmail.com; 2 School of Civil Engineering, VIT Deemed University, Chennai 600127, India; karthikeyank_vit@yahoo.co.in; 3 Department of Civil Engineering, Sri Sairam Engineering College, Chennai 600044, India; sivaramakrishnan.civil@sairam.edu.in; 4 School of Energy and Environment, NICMAR University (National Institute of Construction Management), Pune 411045, India; ssudarsan@nicmar.ac.in; 5 School of Construction Management, NICMAR University (National Institute of Construction Management), Pune 411045, India; 6 Department of Civil Engineering, Wasit University, Kut 52003, Iraq; 7 Peter the Great St. Petersburg Polytechnic University, 195251 Saint Petersburg, Russia; vatin@mail.ru; 8 Division of Research & Innovation, Uttaranchal University, Dehradun 248007, India

**Keywords:** impact strength, reliability, concrete, rubber waste, fibres, Weibull distribution

## Abstract

The proper disposal of used rubber tires has emerged as a primary concern for the environment all over the globe. Millions of tires are thrown away, buried and discarded every year, posing a major environmental concern owing to their slow decomposition. As a result, it is advantageous to use recycled waste rubber aggregates as an additional building resource. Recycling crushed rubber would lead to a long-term solution to the problem of decreasing natural aggregate resources while conserving the environment. This study examines the impact strength variability and reliability of preplaced aggregate concrete containing crumped rubber and fibres. Ten different mixtures were prepared by replacing natural aggregate with crumped rubber (5, 10, 15 and 20%). The crumped rubber was pretreated by the water with sodium hydroxide dilution for 30 min before usage. Hooked-end steel fibres were used at a dosage of 1.5%. The compressive strength, impact strength, impact ductility index and failure pattern were examined and discussed. In addition, a statistical method called Weibull distribution is used to analyze the scattered experimental results. The results showed that when the crumb rubber content was raised, the retained first cracking and failure impact numbers increased. As a result of substituting crumb rubber for 20% of the coarse aggregate in plain and fibrous mixes, the percentage development in first crack and failure was between 33% and 76% and 75% to 129%, respectively.

## 1. Introduction

Concrete is producing considerable use of recyclable and solid waste resources to address environmental challenges and reduce energy consumption [1]. Further research into the production of green concrete is encouraged by the increase in concrete properties and environmental advantages from the use of waste resources [2]. There are several substitutes and solid waste products, such as building debris and demolition [3], plastic wastes [4], rock waste [5], silica fume [6], flyash [7], lime sludge [8] were added to concrete to enhance their properties and minimize emissions of carbon gases while conserving energy [9]. Concrete can be produced using crumpled rubber, which can be derived from used tires used [10]. The manufacturing of automotive tires has substantially expanded due to fast population growth and mobility expansion. Consequently, waste rubber disposal has become a global environmental concern. Around 2900 million new tires are manufactured annually [11], and used tires numbered between 1000 and 1500 million [12]. More than fifty percent of the trash generated by this industry is thrown away or buried without being processed in landfills or rubbish dumps [13]. As a result, the choices of rubbish dumps and landfilling waste tire rubbers are no longer viable due to the diminishing number of available landfill sites and many other environmental issues [14].

Because of such limitations, investigators now have the opportunity to examine alternate applications for waste tire rubbers and develop methods that are more environmentally friendly. In the past, investigators used discarded rubber partially as a coarse aggregate in concrete. When natural coarse aggregate was replaced with rubber, concrete’s compressive and splitting tensile strengths were reduced by roughly 85 and 50% for 100% rubber substitution, respectively [15]. Several investigations have been performed to improve rubber-based concrete’s functionality using various pre-treatment strategies for rubber particles, including physical and chemical processes [16,17]. According to Youssf et al.’s [18] findings, washing rubber with water may result in a marginal improvement in the compressive strength of concrete compared to concrete containing untreated rubber. Another way to increase the rubber-based concrete strength is using cement paste to pre-coat the rubber [19]. However, this technique is not always successful in enhancing concrete strength, when cement paste is used to pre-coating the rubber particles [20]. An additional technique, known as the surface treatment technique, was used to improve concrete functionality. In this technique, the crumpled rubber’s surface was widely used to treat chemicals before the casting process. It is widely accepted that the most efficient pre-treatment approach is NaOH surface treatment [21]. This behaviour is due to using a powerful cleanser, which acts as an agent that increases the surface roughness of rubber. Youssf et al. [22] reported that using pre-treated rubber with NaOH resulted in a 17% improvement in concrete strength compared to untreated rubber-based concrete. Balaha et al. [23] reported that rubber-based concrete with NaOH pre-treated crumpled rubber had a 13% increase in compressive strength. A study reported that treating rubber with a mixture of chemicals consisting of polyethylene glycol, acrylic acid and anhydrous ethanol results in significantly improved workability. Molecular similarities between the modifier and a polycarboxylate-based water reducer are thought to be responsible for the enhanced workability [24]. It was observed by Mohammadi et al. [25] that 24 h of water immersion of rubber aggregates made a substantial difference in the 28-day compressive strength of rubber-to-cement binding because the immersion of rubber particles removes entrapped air from the aggregates. Rivas-Vazquez et al. [26] investigated the results of treating crumb rubber with acetone, methanol and ethanol in a solution that included a solvent concentration equal to 50% of the volume of water. Results revealed that the Ethanol treatment of crumb rubber did not significantly alter the seven-day compressive strength, while the 21 and 28-day strengths exhibited a modest increase compared to the untreated rubber. Acetone was shown to be the most effective treatment strategy, resulting in the greatest increase in compressive strength at 3, 7, 21, and 28 days compared to the untreated and the solvent-treated samples.

Several investigators have reported that rubber-based concrete lacks mechanical properties [18,19,20,22,23]. Fiber incorporation into concrete improved its mechanical properties; hence, the deficiency of rubber-based concrete was alleviated [27,28,29]. According to Alwesabi et al. [30], a significant decrease in concrete compressive strength may be attributed to high crumpled rubber and steel fiber inclusion levels. The drop in flexure and tensile strength caused by the addition of 30% crumpled rubber was mainly compensated for by the improvement in impact resistance provided by the combinations containing 1% SFs. Murali et al. [31] reported that the combined action of steel fibre and crumpled rubber exhibited a higher impact resistance at first crack by about 16.9 to 143.1% for the concrete containing 5–30% of coarse rubber, respectively. Similarly, the impact resistance at the failure stage ranged from 23.5 to 166.2% for the same rubber content range. Liu et al. [32] reported that the optimum rubber content was 10% to exhibit higher impact resistance. However, beyond 10% of rubber content significantly reduced the energy-absorbing capacity. Compared to rubber-based concrete, fiber and rubber-based concrete’s deformation and energy absorption capabilities were improved. Wang et al. [33] reported that the utilization of macro polypropylene fiber and rubber in concrete increases the deformation of concrete’s post-failure and post-failure residual flexural load capacity. Because of this behavior, stress is distributed for the propagation of many cracks, which ultimately increases total fracture toughness and decreases brittleness. Several studies have reported that rubber-based concrete’s impact resistance was increased [34,35,36]. However, the impact test data regarding rubberized concrete with fibres using a drop weight impact test is limited and it required special attention.

In many experiments, fibers and crumpled rubber were shown to have a good influence on concrete’s impact characteristics. As a result, there is no evidence of the creation of preplaced aggregate concrete (PAC) using the crumpled rubber limited from the technical literature. Due to its unique production technique, PAC is a new composite that varies from standard fibrous concrete. Coarse aggregates and fibers were combined in PAC and adequately placed in the framework before grout was injected into the interparticle void region [37]. Small quantities of grout must be fully mixed and pumped during this operation to reduce the effort needed due to the coarser particles and fibres already put in the framework [38]. In order to save time, money, and resources, PAC technology permits thick monoliths to be created without the need for consolidation techniques like vibrations or compactions [39]. This study used the concept of PAC to produce rubber-based fibrous concrete. The impact resistance of rubber-based concrete was evaluated using the drop weight impact test. In addition, the impact results scattering was analyzed using the Weibull distribution method. Finally, the impact strength corresponding to the first crack and breakdown was presented at the reliability level.

## 2. Experimental Program

### 2.1. Materials

**Figure 1:**
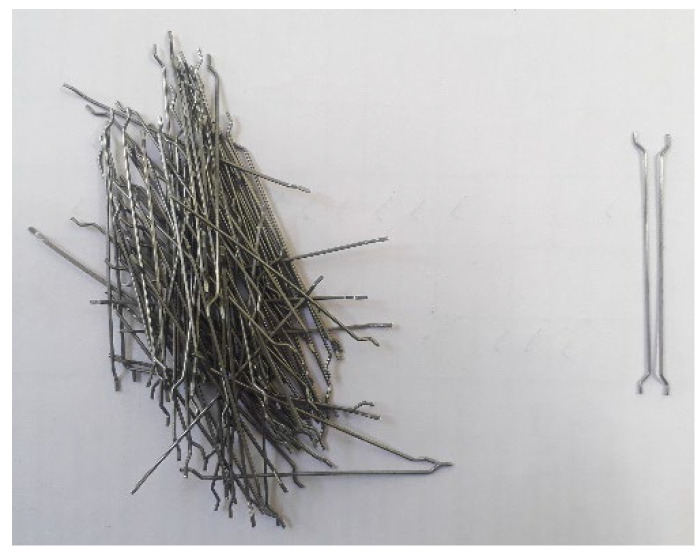
Fibre used in this study.

- According to IS: 12269–1987 [40] requirements, this investigation used an OPC (Ordinary Portland Cement). Its specific gravity was 3.14 and it had a specific surface area of 318 kg/m^2^.
- The fine aggregate was obtained from a natural river that was located nearby. It had a specific gravity of 2.65 and a fineness modulus of 2.41, which is in accordance with the requirements of IS: 383–2016 [41]. In addition, a one-of-a-kind grout mix that conforms to ASTM C939 [42] was validated by employing a fine aggregate particle size less than 2.36 mm. After that, a great gravity flow was accomplished, resulting in effectively filling the voids inside the skeletal aggregate.
- The size of the granite gravel that was utilized for the coarse aggregate was 12.5 mm. The coarse aggregates had a specific gravity of 2.69 and a bulk density of 1700 kg/m^3^, while the percentage of water absorption value was 0.56. To improve the flowability of the grout and satisfy the criteria for the efflux time, a superplasticizer called Tec Mix 640 that is available for commercial purchase was used. The pH value of the superplasticizer that was utilized was between 7 and 8. For the non-fibrous and fibrous specimens, the doses of chosen superplasticizers were 0.3 and 0.6% (by cement weight), respectively.
- The recycled rubber recovered from shredded tires had a density of 700 kg/m^3^. Rubber was sourced from a mechanical shredding plant in Madurai, Tamil Nadu, India. All mixtures were made with crumpled rubber free of steel belts and ranged from 12 to 18 mm.
- Carbon hooked end steel fibre of the corrugated type, which has 1400 MPa tensile strength, a 60-mm length and 0.75-mm diameter, was employed in the concrete at a 1.5% dose. Figure 1 shows the steel fiber used.

### 2.2. Treatment of Rubber

**Figure 2:**
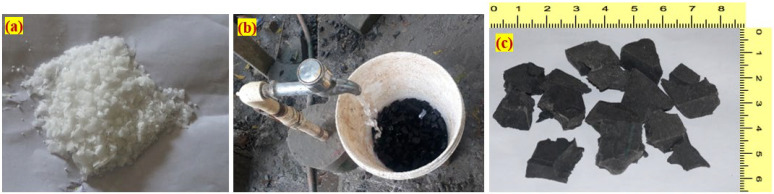
Details of treatment process (**a**) Sodium hydroxide, (**b**) aggregate treated with water containing sodium hydroxide and (**c**) treated rubber.

It is necessary to pre-treat rubber in order to increase its adhesion to the cement. Rubber tires are frequently treated with NaOH solution before use to remove the coating of zinc stearate that has been developed on their surface, rendering them rough and porous [43]. Rubber surfaces that have been treated with NaOH solution have a higher void percentage than those that have not been treated. Since NaOH solution increases hydraulic conductivity, it scrabbles up the rubber surface particles and strengthens the connection between the rubber and the cement [44]. A higher concentration of NaOH intensively etched the rubber, making the surface rougher with more microcracks, providing a larger surface area and higher hydraulic conductivity. The increase in hydraulic conductivity can also be associated with voids created as a result of poor bonding between rubber particles with the concrete composite matrix, which in general can be linked to the increase in porosity as a result of a rise in crumb-rubber content in the concrete composite [44]. In this investigation, the rubber aggregates were first washed thoroughly with tap water to remove any dirt or contaminants that may have accumulated on the surface. For 30 min, the rubber was soaked thoroughly in a 10% NaOH solution. The rubber was then removed from the solution and re-rinsed until it reached a pH of 7, following which it was allowed to dry in the air. Unless the NaOH solution is completely removed, it will have a negative impact on the strength and durability of concrete, necessitating a final cleaning. The NaOH, initial water treatment and treated rubber as shown in Figure 2a–c, respectively.

### 2.3. Combination of Mixing

Ten unique mixtures were created during this experiment, each of which had the same proportions of water-to-cement (*w*/*c*) and sand-to-cement (*s*/*c*). A large number of trial grout mixes were created in order to optimize the water-to-cement and sand-to-cement ratios as well as the amount of superplasticizer that was required to meet the required efflux time (35–40 ±2s) [42]. The detailed mixing combination is demonstrated in Table 1. The natural coarse aggregate was replaced by rubber aggregate up to 20% with every 5% increment. The mixtures were divided into groups A and B. Group A consisted of five mixtures in which the rubber contents used were (0, 5, 10, 15 and 20%). Group B also consists of five mixtures with a 1.5% dosage of fibres and rubber content (0, 5, 10, 15 and 20%). The first mixture is designated as R0 and has zero rubber aggregate content. The letter ‘R’ specifies the rubber concrete and the numeral “0” specifies the percentage of rubber content replaced with natural aggregate. All mixtures from the first group were named using this concept and the specimens without fibres. The rubber content of the second group is the same as the first group except for the 1.5% of the fibres added to all mixtures.

### 2.4. Specimen Preparation

**Figure 3:**
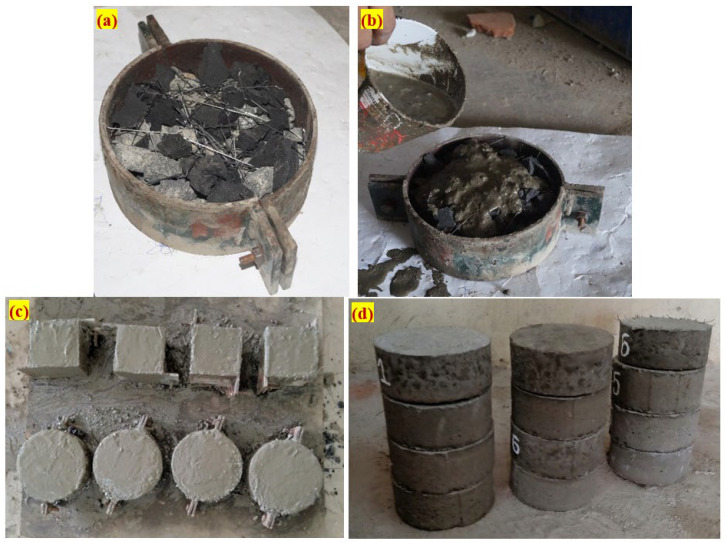
Casting process (**a**) Mold with filled aggregates and fibres (**b**) gravity method of grout injection, (**c**) finished specimens and (**d**) demolded specimens before curing.

The cylindrical disc specimen was fabricated to assess the impact strength of rubber-based concrete. To determine the material’s impact resistance, a specimen in the form of a cylinder was produced and measured 76 mm in radius and 64 mm in thickness. Before the fibres and aggregates could be poured into the mold, the formwork had to be thoroughly cleaned and oil-coated on all inner surfaces. Natural skeletons of aggregates were then created by filling the frameworks with coarse gravel and fibers (Figure 3a). At last, a cement grout with a high flowability was poured on top of the natural skeletons, filling the spaces left between them (Figure 3b). The spaces between the aggregates and the fibers were filled using gravity in this form of grouting; heavy compaction or vibration was not needed at any point in the process. However, some minor compaction was applied to a specimen to prevent honeycombing. The finished specimens after grouting are shown in Figure 3c. The specimens were demolded (Figure 3d) after twenty-four hours. All specimens were cured in water for 28 days prior to testing.

### 2.5. Test Setup

**Figure 4:**
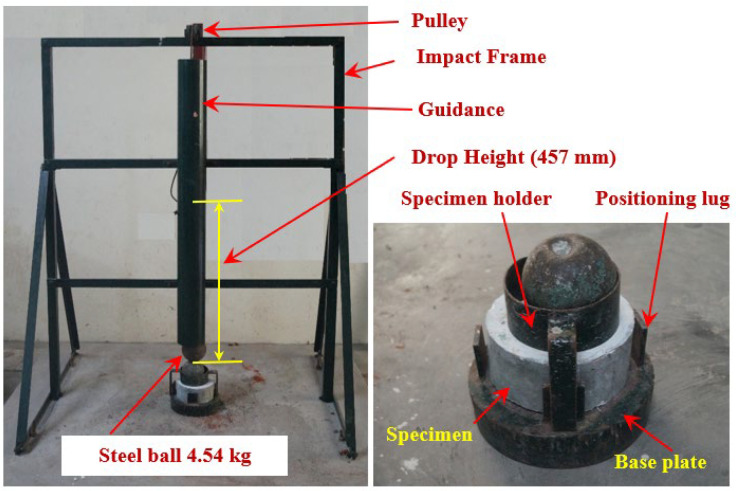
Test setup.

A drop-weight impact test proposed by ACI Committee 544-2R is used to measure the strength of concrete [45]. Impact tests with drop weights are too simplistic since they do not need any load history, deformations, or vibrations. The first crack (T1) and failure (T2) were determined only by counting the number of impacts. The rubber-based concrete specimens were tested for their impact strength using the impact testing instrument shown in Figure 4. The target specimen may be hit by a 4.45 kg weight being dropped from a height of 457 mm using this testing instrument. The elevated steel ball strikes the steel ball positioned on the specimen’s top surface. In order to hold the specimens in place, positioning lugs and a steel disc were utilized in the test. The T1 and T2 impact figures were recorded. The failure is indicated by fractures that begin on the specimen’s top surface and proceed to the bottom. Samples examined visually showed signs of cracking and failure. Each impact delivers the same quantity of impact energy.

## 3. Discussion of Results

### 3.1. Compressive Strength

**Figure 5:**
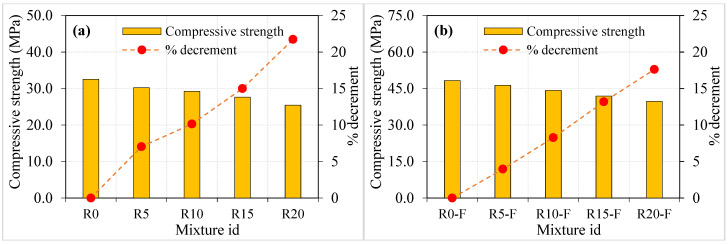
Compressive strength of specimens (**a**) without fibre (**b**) with fibres.

Figure 5 illustrates the rubber-based concrete’s compressive strength with and without fibres. It can be seen from Figure 5 that a reduction in compressive strength was observed with the increasing rubber content. For example, the concrete containing 5, 10, 15 and 20% rubber content resulted in a 7.0, 10.1, 15.0 and 21.73% reduction in compressive strength, respectively, compared to the R0 reference mixture. As indicated in prior research, this tendency could well be ascribed to (a) the weak bond between rubber and mortar facilitating the emergence and spread of microcracks [46]; (b) the surface roughness of rubber particles, which traps air and contribute to an increase in the mixture’s porosity [30]; and (c) the substantial cross-sectional area reduction caused by rubber particles acting as large pores due to the lower stiffness of rubber [47]. The observed results are well aligned with the earlier research [12,48].

Adding fibres to concrete increased the compressive strength from 32.53 to 48.25 MPa, indicating 48.3% improvements. This phenomenon is due to the fibres contributing to concrete’s increased strength by preventing cracks from spreading and enlarging and acting as a bridge mechanism to transmit pressures over the damaged region, and excellent residual strength is observed [49,50]. It can be seen that (Figure 5b), rubber-based fibrous concrete showed a decreasing trend of compressive strength ranging from 46.34 to 39.75 MPa while increasing rubber content from 5 to 20%. Although adding fibres exhibited an excellent compressive strength, the increasing rubber content was not influenced positively. Compared with the R0-F mixture, the compressive strength of R5-F, R10-F, R15-F and R20-F were decreased by 3.9, 8.2, 13.2 and 17.6%, respectively. These reductions in compressive strength were significantly less compared to mixtures without fibres. The combined effect of rubber and fibres positively influenced compressive strength and alleviated the reduction in strength while increasing rubber content. This trend is well-aligned with a former study [31].

### 3.2. Results of Impact Strength

#### 3.2.1. Cracking and Failure Impact Records

**Figure 6:**
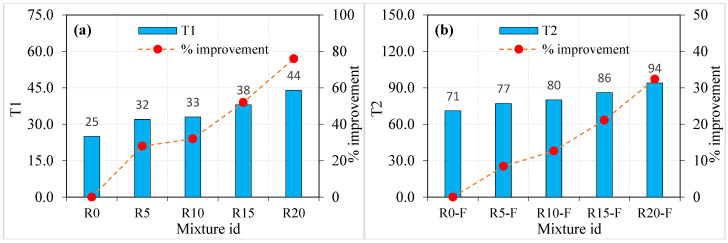
Impact strength of non-fibrous specimen (**a**) T1 and (**b**) T2.

**Figure 7:**
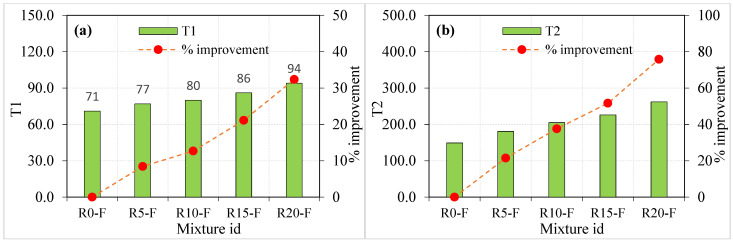
Impact strength of fibrous specimen (**a**) T1 and (**b**) T2.

The obtained experimental results from the ACI 544 [45] repeated impact test are tabulated in Table 2 and Table 3 and depicted in Figure 6 and Figure 7 for both the plain and fibrous mixtures, respectively. The retained results exhibited reasonable scattering compared to what was frequently reported in the literature [51,52], where the COV records at the cracking and failure stages (T1 and T2) of this study were in the range of 12.56 to 27.2%. The reported COV values seem to be high; however, they are lower than those typically reported in the literature [53], where the ACI 544-2R repeated impact test is known for its high results scattering that mostly exceeds 30%.

Three effects can be investigated in the presented impact results of this study, which are the sole effect of crumb rubber, the sole effect of steel fibres and the dual effect of crumb rubber and steel fibres. As is shown in Figure 6a, for the plain mixtures (no fibres), the increasing of crumb rubber led to a continuous increase in the cracking impact resistance. Considering the T1 value of mixture R0 (without rubber) as a reference record, the percentages of increase in T1 due to the incorporation of 5, 10, 15 and 20% of crumb rubber as a partial replacement of aggregate were approximately 26, 32, 32 and 76%, respectively. Similarly, Figure 6b shows that the failure impact resistance also showed a continuous improvement as the percentage replacement of crumb rubber increased. The percentage improvements in T2 of the mixtures R5, R10, R15 and R20 compared to R0 were in the sequence of approximately 36, 52, 85, and 129%. Considering the second group of specimens (Table 3) with steel fibres, it is also obvious in Figure 7 that incorporating crumb rubbers proportionally increased both T1 and T2. The percentage developments due to the incorporation of 5, 10, 15 and 20% of crumb rubber (compared to mixture R0-F) were approximately 8, 14, 22 and 33%, respectively, in T1 (Figure 7a), while they were approximately 21, 37, 51 and 75% in T2, as shown in Figure 7b.

It is obvious from the compressive strength results of the study and in literature [22,23] that crumb rubber decreases the strength of concrete and its modulus of elasticity. In addition to the other reasons discussed in the previous section, this action is mainly attributed to the weak surface bond between the rubber particles and the surrounding cement paste owing to their smooth surfaces. Oppositely, it was recorded in previous studies [54,55,56] that using crumb rubber has the potential of improving the impact resistance, toughness and energy absorption capacity. Al-Tayeb et al. [57] reported that the impact resistance of concrete beams subjected to flexural impacts was enhanced by the partial substitution of sand by crumb rubber. The reported percentage improvements in T1 and T2 for replacement ratios of 5, 10 and 20% were in the ranges of 16 to 35%, 25 to 91% and 50 to 145%, respectively. Khalil et al. [58] reported that percentage increases in T1 and T2 of approximately 80 and 152% were recorded for disk specimens of self-compacting concrete with sand-crumb rubber replacement ratios of 10 and 20%, respectively. Abdel Aleem et al. [30] reported that crumb rubber replacement rations of 5, 10, 15 and 20% by sand in self-compacting concrete increased the flexural impact energy of beam specimens by 21, 68, 101 and 129%, respectively, while the percentage developments for cylindrical impact specimens were approximately 9, 14, 33 and 46%, respectively. The reported percentage improvement ranges show some agreement level with the results obtained in this study. Considering the different rubber particle sizes, different specimen shapes and sizes, different testing boundary conditions and different concrete types tested, it can be generalized that crumb rubber as a replacement of fine or coarse aggregate can noticeably enhance the impact resistance of concrete. Crumb rubber as a low-stiffness material when added to a concrete mixture decreases its stiffness and elastic energy, which negatively affects the mixture’s strength and modulus of elasticity. On the other hand, the high plastic energy capacity and superior ductility of crumb rubber aggregate particles enable them to work as small-size dispersed shock absorbers in the mixture, which results in a tougher and more ductile mixture with a higher impact energy absorption capacity. As a result, the impact resistance of the mixture is increased and its ability to sustain higher impact numbers is improved. 

The effect of steel fibre on the ACI 544-2R repeated impact resistance of different concrete types was extensively investigated in previous studies [39,59,60,61,62,63,64], in which improvement reaching several hundred percentages were reported. The results of this study support this conclusion, where the incorporation of 1.5% of hooked-end SF significantly increased the cracking resistance and boosted the failure impact resistance. The sole effect of SF on T1 and T2 was calculated by comparing the fibrous mixtures (Table 3 and Figure 7) with their corresponding plain mixtures (Table 2 and Figure 6) that have the same crumb rubber quantities; hence, the percentage improvement of T1 and T2 of R10-F due to SF was calculated based on the their increase compared to the corresponding records of R10, and so on for the other mixtures. The percentage developments due to the addition of SF on T1 was found to be in the range of approximately 113 to 183% for the five mixtures, while that of T2 was calculated to be in the range of approximately 326 to 457%. Steel fibres are short high-tensile strength reinforcing elements. Such tiny elements are randomly distributed in the matrix in different directions composing multidirectional discontinuous microstructural reinforcement grids. When a crack is formed inside the matrix under the effect of impact shock waves, it would be restricted by the bridging function of the SF fibres [65]. Hence, the propagation of the initiated cracks to the surface is delayed for several more impact blows owing to SF activity. Thus, the recorded impact blows number that would cause the first surface crack is increased. As soon as the cracks become wider and their number increases after T1 is recorded, the dispersed SF reinforcing elements are being effectively stressed across the cracks trying to prevent its further widening. Owing to the fibres high tensile strength, steel fibres can withstand higher tensile bridging stresses before their rupture or the failure of their surface bond with the surrounding cement matrix. Therefore, the specimens can withstand much higher impact loads before failure, which explains the several times higher T2 records of fibrous specimens compared to the corresponding plain ones [66].

From the previous discussion, it is obvious that the effect of SF was several times higher than the effect of crumb rubber, where the percentage increase due to SF on T1 was approximately 1.5 to 5.5 times that due to crumb rubber for the same mixtures. Similarly, the percentage improvements in T2 due to the addition of SF were approximately 2.5 to 11 times those due to the incorporation of crumb rubber. The different effects are attributed to the different functions of the two materials, where the first helps increase the capacity of the concrete mixture to absorb higher impact shocks, which is decreased after cracking and diminishes when the bond between the crumb rubber particles and the surrounding matrix is diminished. On the other hand, the cracking bridging activity of SF lasts even after the propagation and widening of the cracks due to the high bond and tensile strengths of the fibres. The dual action of both materials is expected to result in accumulated improvements that are higher than their individual effects. This result was obtained in this study, where by comparing T1 and T2 records of the fibrous specimens with those of the reference mixture R0 that includes neither crumb rubber nor SF, their dual effects can be measured. For the mixtures R5-F, R10-F, R15-F and R20-F that include 1.5% of SF and 5, 10, 15 and 20% of crumb rubber, the percentage improvements in T1 compared to that of R0 were calculated to be approximately 206, 221, 245 and 275%, respectively. On the other hand, the corresponding percentage developments in T2 were approximately 573, 662, 742 and 876%, respectively. Thus, it can be concluded that it is recommended to use preplaced concrete mixtures with 20% crumb rubber replacement and 1.5% SF where the main purpose of the designed structural element is to absorb low-velocity impact shocks. 

#### 3.2.2. Impact Ductility

**Figure 8:**
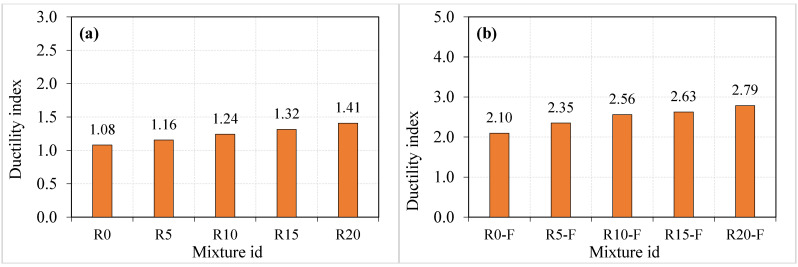
Impact ductility index of specimens (**a**) Non fibrous and (**b**) Fibrous.

The term ductility refers to a physical quantity that is usually used to define the ability of flexural members to absorb plastic energy. The plastic region in the load-displacement curves of members under bending loads is calculated for the post-yielding region, which starts from the yielding point of the tension reinforcement to failure. This definition was also facilitated to investigate the effect of fibres and other additives on the post-cracking behaviour of disk specimens subjected to the ACI 544-2R repeated impact, where several previous studies [67,68,69,70] defined the impact ductility index as the ratio of the failure number to the cracking number (T2/T1). This study also adopted this definition to evaluate the influence of crumb rubber and SF on the enhancement of the failure impact resistance. Hence, the higher the ratio of T2/T1, the more ductile the mixture is. Figure 8a shows clearly that for plain specimens, the impact ductility index increased with the increase of crumb rubber percentage replacement, where the incorporation of 5, 10, 15 and 20% of crumb rubber increased the ductility index by approximately 7, 15, 22 and 31%, respectively, compared to that of the reference mixture R0. Similarly, for fibrous specimens, Figure 8b explicitly reveals a continuous increase in the ductility with the increase of crumb rubber replacement ratio, where percentage developments in the impact ductility of approximately 12, 22, 25 and 33% (compared to mixture R0-F) were recorded as 5, 10, 15 and 20% crumb rubber replacements were utilized. The increase of the mixture ductility is due to the better ductility and plastic energy capacity of rubber [30,71].

On the other hand, the capacity of SF to increase the impact ductility is higher than that of crumb rubber, where the effect of SF can be calculated by comparing the ductility indices of fibrous specimens to their corresponding plain specimens that have the same crumb rubber quantities. The percentage developments in the impact ductility due to SF only were in the range of approximately 94 to 106%, which is much higher than that obtained due to crumb rubber. This result is attributed to the main function of SF as crack bridging elements that become fully functional after cracking, which boosts the failure impact numbers compared to their corresponding cracking numbers, resulting in higher ductility [70]. Finally, the combined effect of SF and crumb rubber can be calculated by comparing the ductility indices of the fibrous mixtures R5-F, R10-F, R15-F and R20-F with the ductility index of the reference plain mixture R0. The calculated percentage developments of these mixtures were approximately 118, 137, 143 and 158%. Thus, it is again proved that the dual action of SF and recycled crumb rubber is a suitable low-cost solution to improve the impact energy absorption capacity and the ductility of structural members subjected to low-velocity impact. Poor interfacial bonding between the cement paste and the rubber aggregate is caused by microcracks on rubber aggregate lowering the mixture’s mechanical strength [47,48,72]. Rubber-based concrete has a larger porosity due to the inclusion of rubber particles, which increases the specific surface area of the concrete and causes poor bonding [73]. Similar findings were found for the rubber-cement paste interface by Emiroğlu et al. [74]. In addition, the combination action of flexible rubber aggregate and steel fibers may be responsible for the improved impact performance ductility index of rubber-based concrete. The steel fibre is responsible for bridging action and rubber aggregates absorb impact energy efficiently. Rubber concrete, on the other hand, resulted in a somewhat weak interfacial bonding, which may be decreased by the fiber bridging action.

#### 3.2.3. Failure Pattern

**Figure 9:**
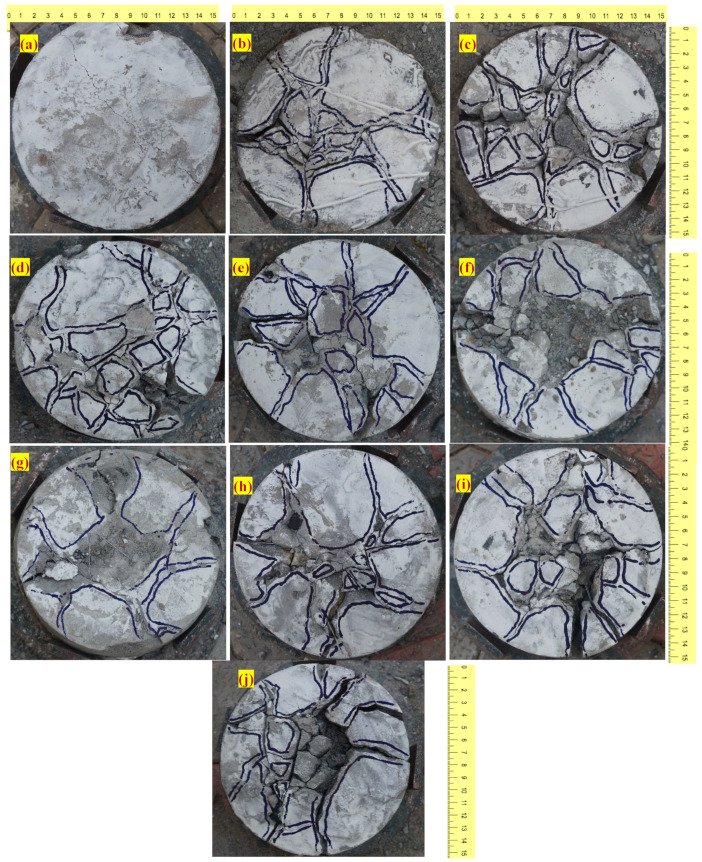
Failure pattern of specimens under impact loading. (**a**) R0, (**b**) R5, (**c**) R10, (**d**) R15, (**e**) R20, (**f**) R0-F, (**g**) R5-F, (**h**) R10-F, (**i**) R15-F and (**j**) R20-F.

The typical failure modes of rubber-based concrete with and without fibre are illustrated in Figure 9. When crumped rubber was added, the failure pattern was altered from a single large crack (Figure 9a) to many intersecting tiny cracks (Figure 9b–e). Adding fibers and rubber to the mixtures seems to increase the number of cracks while decreasing crack breadth, as seen in Figure 9g–j. As a consequence of the activity of the fibres, the specimens containing rubber aggregate and fibres had tiny fractures, which prevented the specimens from collapsing unexpectedly. Observations of the behavior of these specimens demonstrated that they might maintain structural integrity while also being ductile. Steel fibres, in combination with rubber aggregates, dramatically modified its failure mechanism from single fractures to numerous cracks, which is a ductile failure, and so the favorable effect of a specimen under impact loading was shown by these failure patterns [30,71]. Consequently, the positive effects of specimens subjected to impact loading were shown in Figure 9. There is a slight depression in the specimens’ surface area where they were hit, which was seen to have shriveled. Impact energy is absorbed and dissipated by de-bonding, sliding, and pulling fibers from the matrix, which results in a rise in impact energy that correlates to failure [75,76].

#### 3.2.4. Failure Mechanism

**Figure 10:**
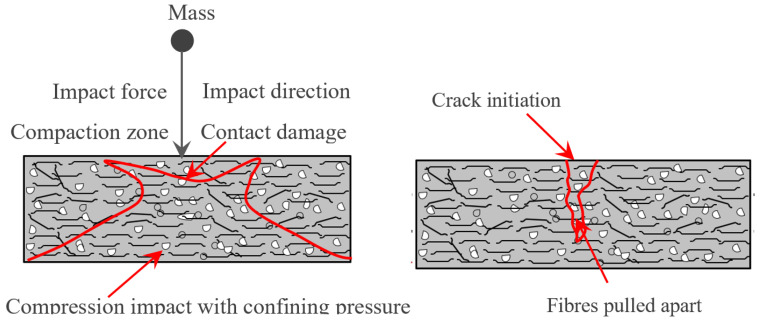
Failure mechanism under impact loading.

Under impact loading, the most common mechanisms for rubber based concrete fracture are cracking, shearing, and compaction. Compression, tension, or restricting pressures may cause the concrete to fracture in the direction of the forces exerted. Nevertheless, the production of eventual fractures in the complete concrete specimen depends on the concrete’s essential properties, which include fibres and crumpled rubber. Consequently, after the first fracture has developed, the fibres can still bridge it and disperse the energies to multiple adjacent areas inside the concrete. At the same time, the presence of rubber absorbs the higher amount of impact energy together with the fibre bridging action. Concrete is damaged when the fibers can no longer inhibit cracks from occurring and are pulled out by the impact stress distribution. Contact damage, fiber rupturing, matrix failure, fiber delamination, loss of bonding between the rubber aggregate and matrix when specimens were exposed to impact testing (Figure 10). The phenomenon of damage to the specimen due to direct collision. Compressive bending on the impact plane ultimately causes the matrix fibres to fail. Fibre debonding advances to neighboring locations due to tensile bending at the bottom surface. Finally, weak bonding between the rubber and matrix results in internal failure. Fibers are separated from their matrix in a critical phase in the failure process, which severely influences the material’s strength.

## 4. Statistical Analysis—Weibull Distribution

The Weibull distribution of two-parameters is widely documented as a suitable model for industrial sector design purposes [77]. Using a systematic review, Jung and Schindler [78] reviewed forty-six studies published between 2010 and 2018 with various theoretical parametric distributions. According to the review results, the distribution of two-parameter Weibull considerably evaluated all the distributions studied. Neha et al. [79] compared the density functions of probability that were applied to impact strength data and discovered that the Weibull distribution of two parameters had several advantages including (i) simplicity in parameter estimation despite the method used; (ii) usage of two parameters alone; (iii) flexibility, etc. In the early stages of impact strength assessment, the researchers observed that drop weight impact test results were unreliable. Although a significant amount of work was put forward to ensure that performance parameters were the same across all participants, the outcomes were inherently incompatible. As a result, it was preferred to employ mathematical formalism to analyze the scattered test results. The impact strength of several studies reported in bibliographic records followed normal distribution [80] and the Weibull distribution [81]. A helpful tool in the context of brittle materials is the probability density function of Weibull, which considers adequate safety strength in the context of reliability. According to the theory of weakest-link, Weibull distribution is named after Swedish mathematician Waloddi Weibull, who worked to clarify in detail the distribution of probability. Weibull [82] noted a significant deviation from the distribution of Weibull earlier in his career. His case studies represent the most critical problems which arose up to that point. Weibull’s concepts were refined mathematically due to his subsequent work. Most variability was caused by non-homogeneity, characterized by several inaccuracy distributions that follow the standard Weibull model to a greater or lesser extent. When describing impact strength data observed in this study, two-parameter distributions of the Weibull distribution were used for the sake of simplicity. *T_f_* can be expressed as impact strength distribution using the Weibull function f(L_f_), found in [83].

(1) $$f\left(T_{f}\right)=\frac{b}{T_{a}-T_{0}}{\left[\frac{T_{f}-L_{0}}{T_{a}-T_{0}}\right]}^{b-1}exp\left\{-{\left[\frac{T_{f}-T_{0}}{T_{a}-T_{0}}\right]}^{b}\right\}\left(T_{0}\leq T_{f}<\infty \right)$$

where *b* is the Weibull slope which describes a shape parameter (distribution variability), *T_a_* describes the Weibull strength and scale parameter, *T*_0_ describes impact strength corresponding to minimum safety, *T_f_* describes a Weibull variable (impact strength). Function f(K) is derived from Equation (1) and expressed in Equation (2) as follows [84]:

(2) $$f\left(T_{p}\right)=P\left(T_{f}<T_{p}\right)=1-exp\left\{-{\left[\frac{T_{p}-T_{0}}{T_{a}-T_{0}}\right]}^{b}\right\}$$

If *P* K_f_ < K which describes a failure probability, then:

(3) $$P\left(T_{f}>T_{p}\right)=1-P\left(T_{f}<T_{p}\right)=exp\left\{-{\left[\frac{T_{p}-T_{0}}{T_{a}-T_{0}}\right]}^{b}\right\}$$

Equation (3) helps achieve *T*_0_, *T_a_* and *b* if all are identified within a specified survival rate *P*. Due to the separate impact strength of the composite, *T*_0_ of concrete with and without fibres was considered zero [84]. Therefore, Equation (1) can be simplified to Weibull’s two-parameter functions as:

(4) $$f\left(T_{f}\right)=\frac{b}{L_{a}}{\left[\frac{T_{f}}{T_{a}}\right]}^{b-1}exp\left\{-{\left[\frac{T_{f}}{T_{a}}\right]}^{b}\right\}\left(T_{0}\leq T_{f}<\infty \right)$$

Failure and survival probability can be expressed in the following Equation:

(5) $$p=exp\left\{-{\left[\frac{T_{f}}{T_{a}}\right]}^{b}\right\}$$

(6) $$p^{\prime }=1-p=1-exp\left\{-{\left[\frac{T_{f}}{T_{a}}\right]}^{b}\right\}$$

If a natural logarithm is considered with two sides in Equation (5), the obtained expression is as follows:

(7) $$ln\left[ln\left(\frac{1}{p}\right)\right]=blnT_{f}-blnT_{a}$$

Rearranging Equation (7) in linear form, it is expressed as follows,

Let Y = $ln\left[ln\left(\frac{1}{p}\right)\right]=ln\left[ln\left(\frac{1}{1-p\prime }\right)\right],\;X=lnT_{f},\;a=blnT_{a}$, then the expression obtained in the form of regression linear can be expressed as follows:

(8) Y=bX−a

**Figure 11:**
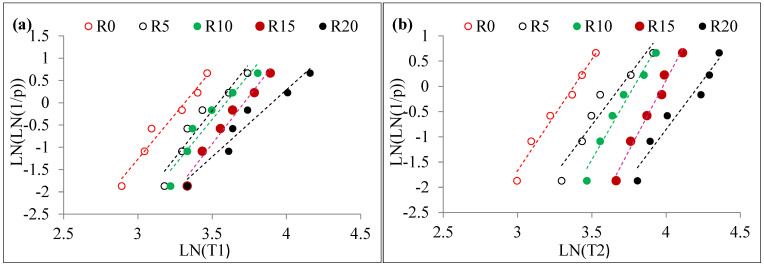
Weibull lines for non-fibrous specimens (**a**) T1 and (**b**) T2.

**Figure 12:**
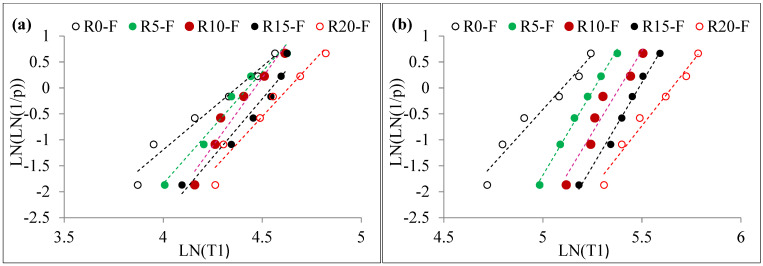
Weibull lines for Fibrous specimens (**a**) T1 and (**b**) T2.

Weibull functions define the impact strength equations given above, and can be written by using the linear function of Equation (8). It is possible to determine whether impact test data was distributed using the Weibull two-parameter distribution. Based on the impact results tests, a straightforward linear regression analysis was performed. It was demonstrated that test data confirmed the distribution of the two-parameter Weibull when an excellent linear correlation between *Y* and *X* was obtained. The following relationship can be used to determine the relationship between L_f_ and *p*:

(9) $$p=1-\frac{i}{k+1}$$

where describes an ascending order of impact strength data points and *k* describes the tested specimen’s number per mixture. As shown in Figure 11 and Figure 12, a graph is plotted between ln[ln(1/*p*)] for each test data point with the corresponding impact numbers ln(T1 or T2). Assuming that data points are linearly correlated, it is reasonable to conclude that impact strength information follows the two-parameter distribution of Weibull. The shape parameter is obtained directly from the slope of the line derived from the linear equation, whereas the scale parameter is derived from Equation (10) as follows.

(10) $$L_{a}=e^{\left(-a/b\right)}$$

Weibull parameters were evaluated and used to assess impact strength that correspond to the necessary reliability level, and are expressed in Equation (11) [85] as a function of the scale parameters.

(11) $$T_{1,2}=T_{a}(-\ln{\left(R_{x}\right)}^{\left(1/b\right)}$$

The results of the linear regression analysis of the specimen are shown in Table 4, which reveals that data subject to the tests was distributed linearly. Furthermore, the determination coefficient (R^2^) of the fitting results for all tested data was between 0.9 and 1.0 in all cases. This clearly shows that *Y* and *X* are linearly dependent on one another. In other words, impact strength follows the two-parameter distribution of Weibull’s law of large numbers.

**Figure 13:**
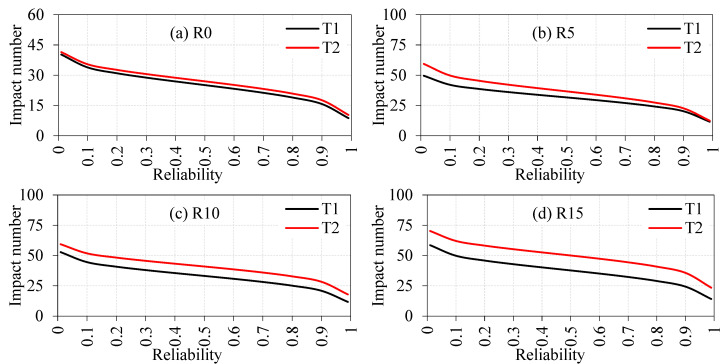

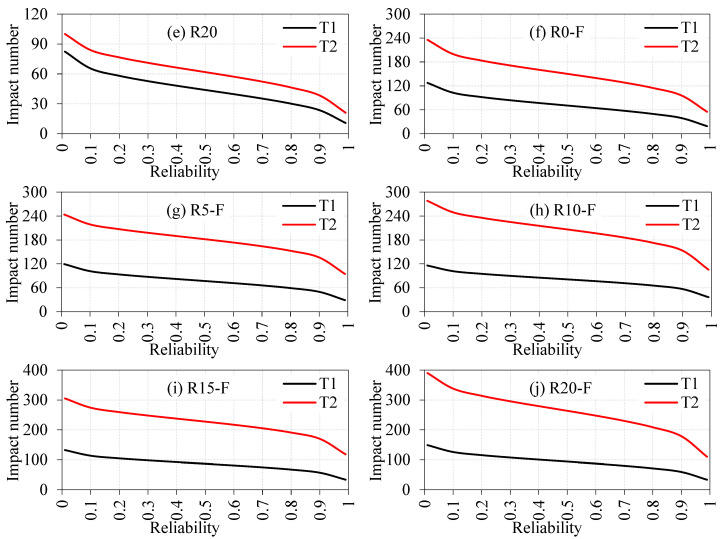
Impact strength in terms of reliability (**a**) R0, (**b**) R5, (**c**) R10, (**d**) R15, (**e**) R20, (**f**) R0-F, (**g**) R5-F, (**h**) R10-F, (**i**) R15-F and (**j**) R20-F.

The calculated Weibull parameters were used to assess the reliability of impact numbers T1 and T2. Table 4 demonstrates the assessed T1 and T2 from the reliability analysis. By reviewing the R0 specimen with 0.99, 0.9, 0.8, 0.7, 0.6 and 0.5 reliability levels, the T1 values were 9, 16, 19, 21, 23 and 25, respectively. Likewise, T2 for the same mixture specimens was 10, 18, 21, 23, 25 and 27, respectively. The obtained R^2^ values for T1 and T2 for this mixture was above 0.9. Impact strength (T1 and T2) for the other mixtures of concrete specimens can be derived from Table 4. R^2^ values for all specimens was above 0.9, which showed an effective fit linear regression. Reliability analysis with the aid of Weibull distribution was used to analyze differences in the experimental testing dataset appropriately. This eliminated the money and effort of evaluating the impact strength of the rubber-based concrete specimens through the drop weight test. A designer can select the impact strength from Figure 13, suggesting strength reliability as a function of reliability for engineering calculations. The results of this study are consistent with past findings [86,87]. It offers reinforced concrete material manufacturers a tool that allows them to display the mechanical properties needed with assurance to the end-user.

Table 5 demonstrates the comparison of results obtained from experimental and statistical analysis. Since drop weight impact experimental results are unreliable, statistical analysis is essential to clarify the variations in test results. Considering the reliability of 0.99, the impact numbers obtained from statistical analysis are shown in Table 5, which are significantly less than the experimental values. The impact strength values are taken from the reliability curves with required reliability function for the design calculations. It is essential to highlight that experimental and statistical analysis results should not coincide due to the nonreliable test results.

## 5. Conclusions

This study examines the combined effect of steel fibre and rubber aggregate in concrete under drop weight impact. The four different percentages of rubber content (5, 10, 15 and 20%) with constant fibre dosage were used to produce specimens and tested. Based on the findings obtained from the research, the following conclusions may be inferred.

- The compressive strength of the rubber-based fibrous concrete decreased from 46.34 to 39.75 MPa as the rubber content increased from 5% to 20%. Compared with the R0-F mixture, the compressive strength of rubber-based concrete was decreased by 3.9, 8.2, 13.2 and 17.6% when the corresponding rubber content was 5, 10, 15 and 20%, respectively.
- Owing to their high plastic energy capacity and superior ductility, crumb rubber particles work as randomly distributed shock absorbers in the mixture, which results in a tougher and more ductile mixture with higher impact energy absorption capacity. As a result, the retained cracking T1 and failure T2 impact numbers were increased as the content of crumb rubber was increased. The percentage developments in T1 and T2 due to the substitution of 20% of the mixture’s coarse aggregate by crumb rubber were in the ranges of approximately 33% to 76% and 75% to 129%, respectively, for plain and fibrous mixtures.
- Steel fibre was found to be a better impact resistance enhancer compared to crumb rubber. The percentage developments due to the sole effect of SF were in the ranges of approximately 113% to 183% for T1 and 326% to 457% for T2, for all crumb rubber ratios. The better effect of SF is mainly due to the crack bridging activity that continues even after crack propagation and widening, which is attributed to the fiber’s high tensile strength and the superior bond with the surrounding matrix.
- The dual action of crumb rubber and SF was found to be very effective in achieving the best impact resistance enhancement results. The percentage improvements of the fibrous rubberized mixtures compared to the reference plain mixture that includes no rubber ranged from approximately 206% to 275% for T1 and 573% to 876% for T2. This gain represents the summation of the positive actions of both materials as shock absorbers and crack bridging elements.
- The impact ductility was increased by the incorporation of crumb rubber and was better improved by the addition of 1.5% of hooked-end SF, while the dual action of the two materials resulted in the highest ductility improvement percentages. The use of 20% of crumb rubber increased the ductility index by approximately 31% to 33%; the used 1.5% of SF increased the ductility index by approximately 98% to 106%, while a percentage improvement range of approximately 118% to 158% was achieved by their combined effect. Additionally, the poor interfacial bonding between the rubber aggregate and cement matrix is due to a larger porosity in rubber aggregates and the increased specific surface area of the concrete. This effect can be minimized by the bridging action of steel fibres that resulted in the higher ductility index of concrete.
- Impact numbers for T1 and T2 in terms of the required level of reliability were evaluated from the Weibull distribution, which can be accepted as a useful statistical analysis approach to determine the impact resistance of specimens without additional expensive and time-consuming tests. The Weibull distribution is an excellent choice to elucidate scattered test results.

## Figures and Tables

**Table 1:** Mixing combination.

Group	S. No	*W*/*C*	*s*/*c*	Natural Aggregate (%)	Rubber Content (%)	Fibre Dosage (%)	Superplastizer (%)
	R0	0.42	1.0	100	0	0.0	0.4
	R5			95	5	0.0	0.4
A	R10			90	10	0.0	0.4
	R15			85	15	0.0	0.4
	R20			80	20	0.0	0.4
	R0-F			100	0	1.5	0.6
	R5-F			95	5	1.5	0.6
B	R10-F			90	10	1.5	0.6
	R15-F			85	15	1.5	0.6
	R20-F			80	20	1.5	0.6

**Table 2:** Impact test results for non-fibrous specimens.

S. No	R0		R5		R-10		R15		R20	
	T1	T2	T1	T2	T1	T2	T1	T2	T1	T2
1	18	20	24	27	25	32	28	39	28	45
2	21	22	27	31	28	35	31	43	37	49
3	22	25	28	33	29	38	35	48	38	55
4	27	29	31	35	33	41	38	53	42	69
5	30	31	37	43	38	47	44	54	55	73
6	32	34	42	50	45	51	49	61	64	78
Mean	25	27	32	37	33	41	38	50	44	62
SD	5.0	4.9	6.2	7.7	6.8	6.6	7.2	7.3	12.0	12.5
COV (%)	20.13	18.44	19.63	21.21	20.48	16.23	19.28	14.69	27.30	20.26

SD: Standard deviation, COV: coefficient of variation.

**Table 3:** Impact test results for fibrous specimens.

S. No	R0-F		R5-F		R10-F		R15-F		R20-F	
	T1	T2	T1	T2	T1	T2	T1	T2	T1	T2
1	48	112	55	146	64	167	60	178	71	202
2	52	121	67	162	71	189	77	209	74	221
3	64	135	73	174	73	193	86	221	89	242
4	76	161	77	186	82	201	94	233	95	276
5	88	178	85	199	91	231	99	246	109	306
6	96	189	102	216	101	246	102	268	124	325
Mean	71	149	77	181	80	205	86	226	94	262
SD	17.7	28.7	14.6	23.1	12.6	26.5	14.4	28.4	18.6	44.3
COV (%)	25.03	19.20	19.15	12.81	15.67	12.96	16.69	12.56	19.87	16.91

SD: Standard deviation, COV: coefficient of variation.

**Table 4:** Weibull parameters.

Mixture ID	T1/T2	b	Intercept	T_a_	R^2^
R0	T1	4.02	−13.31	27.52	0.963
	T2	4.41	−14.91	29.32	0.978
R5	T1	4.23	−14.97	34.55	0.924
	T2	3.93	−14.53	40.26	0.925
R10	T1	4.07	−14.63	36.30	0.922
	T2	5.11	−19.34	44.00	0.964
R15	T1	4.30	−9.98	41.05	0.972
	T2	5.58	−22.20	53.43	0.982
R20	T1	3.01	−11.74	49.57	0.942
	T2	3.93	−16.58	67.84	0.945
R0-F	T1	3.21	−14.02	79.19	0.961
	T2	4.21	−21.48	163.81	0.955
R5-F	T1	4.31	−19.10	83.75	0.972
	T2	6.47	−34.04	192.47	0.995
R10-F	T1	5.28	−23.57	86.74	0.949
	T2	6.32	−34.02	218.43	0.940
R15-F	T1	4.50	−20.48	94.32	0.951
	T2	6.45	−35.37	240.90	0.989
R20-F	T1	4.13	−19.13	102.89	0.946
	T2	4.84	−27.36	284.51	0.971

**Table 5:** Comparison of experimental value with statistical analysis value.

Mixture ID	T1		T2	
	Experimental	Statistical (0.99 Reliability)	Experimental	Statistical (0.99 Reliability)
R0	25	9	27	10
R5	32	12	37	13
R10	33	12	41	18
R15	38	14	50	23
R20	44	11	62	21
R0-F	71	19	149	55
R5-F	77	29	181	95
R10-F	80	36	205	105
R15-F	86	34	226	118
R20-F	94	34	262	110

## Data Availability

Not applicable.
